# Principal Component Thermography for Defect Detection in Concrete

**DOI:** 10.3390/s20143891

**Published:** 2020-07-13

**Authors:** Bojan Milovanović, Mergim Gaši, Sanjin Gumbarević

**Affiliations:** Faculty of Civil Engineering, Department of Materials, Fra Andrije Kačića Miošića 26, University of Zagreb, 10000 Zagreb, Croatia; mgasi@grad.hr (M.G.); sgumbarevic@grad.hr (S.G.)

**Keywords:** infrared thermography, concrete, principal component analysis, non-destructive testing, defect detection

## Abstract

The goal of the condition assessment of concrete structures is to gain an insight into current condition of concrete and the existence of defects, which decrease durability and usability of the structure. Defects are quite difficult to detect using infrared thermography when concrete elements cannot be thermally excited with the Sun, together with unfavorable thermophysical properties of concrete structures. In this paper, principal component thermography (PCT) is applied as a post-processing method to a sequence of thermograms in order to enhance defect detectability in concrete structures. Defects are detected by analyzing a set of empirical orthogonal functions (EOFs), which were acquired by applying principal component analysis to a sequence of thermograms. The research was performed using concrete samples containing known defects, which were tested using a step heating thermography setup. The results of presented research show that PCT is an effective post-processing method to improve defect detection in concrete structures. By effectively improving the defect detection, PCT has a potential to improve the non-destructive testing (NDT) accuracy of using infrared thermography (IRT) on concrete structures, especially in shaded areas of such structures. The research also shows the defect detectability depending on concrete type thermal excitation setup and defect geometry.

## 1. Introduction

Today concrete is understood to be one of the most widely used materials in civil engineering structures around the world [1,2]. Concrete is also considered to be a strong and robust material, which can endure significant external degradation mechanisms [3]. On the other hand, concrete is by its nature a nonhomogeneous composite material whose composition varies and a material that utilizes diverse raw materials in its production. This causes quite a lot of complications when one tries to apply non-destructive testing (NDT) methods to detect defects in concrete structures. Additionally, besides precast concrete elements produced in a concrete plant, usually ready-mix concrete is being produced in small concrete plants and then delivered and cast in-situ. Since work on in-situ casting is often performed by unskilled workers, the resulting hardened concrete is highly variable and often contains defects, which can influence its durability. This means that concrete structures are often not constructed in a way that would meet the designed parameters [4].

The goal of the condition assessment of concrete structures is to gain an insight into current condition of the concrete, the existence of voids, delamination and other defects. Detection and characterization of different defects in concrete structures is often performed using one or (more often) several different reliable and efficient NDT methods, which have been developed over the last 30 years. These methods serve different purposes, from corrosion detection methods, methods used to determine mechanical properties and permeability of concrete, up to a vast range of methods and techniques used for detection and characterization of defects and damaged areas in new and existing concrete structures [5,6,7,8].

To date, the biggest advantage of using infrared thermography (IRT) for defect detection in concrete structures was its ability to perform an assessment of large surfaces in relatively short period of time [9], and to pinpoint defective areas. These defective areas would then be further analyzed and defects quantified using other NDT or even semi-destructive methods. Some researchers [10] state that methods like ground penetrating radar, ultrasound and sonic methods are mostly suitable for detection and characterization of inhomogeneous areas with a depth of more than 5 to 10 cm, while active IR thermography is useful for testing the shallow regions up to 10 cm in depth.

Passive IRT as a method used for defect detection in concrete structures is based on quite simple principles and are fairly straightforward to carry out if the concrete structure to be tested is under natural, solar excitation (unobstructed by buildings, trees, or other objects). Passive IRT is being used for concrete bridge deck testing since the late 1970 s [11,12] and is being successfully utilized in combination with other NDT methods for bridge deck testing today [9,13,14,15].

On the other hand, active IRT is done by utilizing some sort of thermal excitation of the object under examination and by simultaneous monitoring of object’s surface temperature evolution in time, especially when the object is in transient state. In solids, thermal energy is diffused in a form of thermal wave, while the IR camera is used to monitor the temporal change of temperature field on the object’s surface [16]. Defects embedded in concrete structures are usually filled with air or in some cases with water and thus have different thermal properties (thermal conductivity and thermal capacity) compared to sound areas of concrete. Heat propagation through the structure is affected by the defects. Detection of these defects using IR camera is possible if the difference in thermal properties between sound and defective areas of concrete is sufficient, and if NDT is performed in transient conditions.

Defect quantification (determination of their size, thickness and embedment depth) in concrete structures using IRT was performed by only a few research groups [10,17,18,19,20]. From the literature review, it can be said that researchers are using impulse-thermography (IT), pulse phase thermography (PPT) and lock-in thermography (LT) in their efforts to detect defects in concrete structures and/or concrete samples. They are quite successful in detecting large defects and moderately successful in characterization of large defects primarily in determining the defect’s depth and their results are acceptable only in controlled laboratory conditions. Post-processing techniques, which are being used by majority of researchers, are fast Fourier transformation [21], singular value decomposition analysis [22], correlation operator’s technique [23]. An extensive review of the use of active IRT for detection and characterization of defects in reinforced concrete is published in [24].

If one considers real conditions in-situ, detection of defects using IRT is hindered by the solar irradiance variations, shading caused by clouds or the surrounding objects, as well as the condition of the concrete’s surface (color variations, texture and possible rubble) [25,26,27]. The problem also emerges when IRT is intended to be used in shaded areas or concrete elements that cannot be naturally thermally excited by the Sun. In such situations, researchers have employed active IRT and used artificial thermal excitation systems like lamps [28], microwave heating [29], etc. 

Common conclusion from the review of the literature dealing with active thermography in concrete structures is that in case of artificial thermal excitation, defects are quite difficult to detect. This is due to thermal inertia of concrete structures owing to specific heat capacity and density of concrete as well as considerable dimensions of concrete structures, while at the same time, concrete has relatively low thermal conductivity. It means that significant amount of energy is needed to influence the temperature change and to initiate a thermal wave propagation in concrete structures. Additionally, when using artificial thermal excitation sources, the problem with non-uniform heating occurs. This means that often, defects are masked by the temperature field caused by focused thermal excitation source, which can lead to false positive IRT inspection results. 

When all these surface and subsurface effects are combined with the surroundings affecting the IRT thermograms, straightforward interpretation of thermograms turns out to be challenging, if not even impossible in some cases. Thus, in order to be able to enhance defect detectability in concrete by using active IRT, complex post-processing methods are required for thermogram analysis. 

One of the post-processing methods that could be used for defect detectability enhancement is principal component analysis (PCA), which is not studied enough when it comes to defect detection in concrete structures. Even though it has a mature background in enhancing thermal pattern in metals and fibre-reinforced composite materials [30,31,32], research dealing with defect detection using principal component thermography (PCT) in concrete structures is scarce [22,24,33]. Furthermore, literature review showed that authors have more success in cases when natural excitation was used, while in case of using artificial excitation the detection of defects using PCT in concrete proved to be difficult. PCT is a post-processing method founded on the concept of PCA and it is used in the presented research since there are research reports [34] which state that results are unaffected by surface effects like non-uniform heating or changes in the emissivity. 

The aim of the presented research was to determine whether the post-processing method of PCT could be applied to data obtained with step heating thermography to enhance the defect detectability in concrete structures. Additionally, the paper explores more deeply the possibilities and limitations of the PCT and gives insight in defect detectability depending on the defect dimensions, properties of concrete and thermal excitation distance. This would then serve to broaden the current knowledge on the defect detection possibilities in concrete using IRT.

## 2. Principal Component Thermography

PCA is a statistical tool used for identification of specific patterns in data sets, and analyzing data sets in a way which enables depicting similarities and differences of specific patterns occurring in the data using statistical modes acquired by decomposing data to singular values [35]. The use of PCA in post-processing thermographic data was proposed by Rajic [31] and the technique was called PCT.

**Figure 1:**
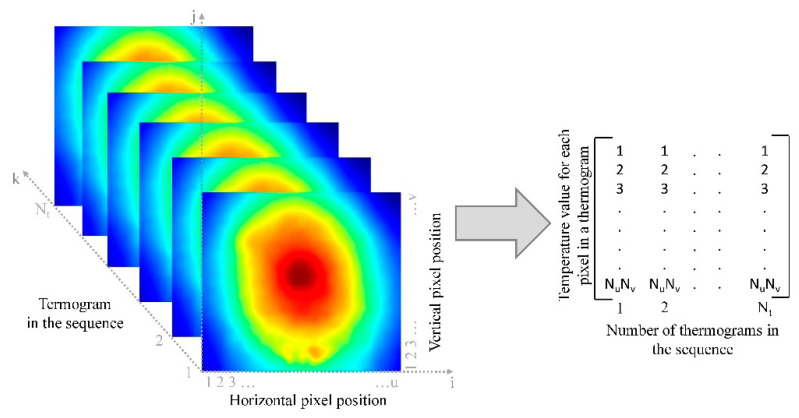
Formation of a 2D raster-like matrix from a thermogram sequence.

The key idea of the PCT is to transform the time-based information to a new domain where temperature variation detected by the series of thermograms might be associated with defects within the structure. In doing so, the thermogram sequence can be considered as a 3D matrix consisting of N_t_ thermograms, each thermogram consists of *u* horizontal elements (pixels)—N_u_ and *v* vertical elements (pixels)—N_v_ (Figure 1). 

In order to apply PCT to a thermogram sequence, it is necessary to convert the 3D matrix to a 2D matrix, which is achieved by the following procedure: Each thermogram in the sequence can be represented by a N_u_∙N_v_ dimensional vector **X** (Equation (1)):

(1) $$X=\left[x_{1},x_{2},x_{3},\ldots ,x_{N_{u}\cdot N_{v}}\right],$$

where x_i_ are the individual pixels of the thermogram arranged one behind the other thus forming a 1D vector.

If an analogous procedure is followed with all N_t_ thermograms in the sequence, it is possible to generate a 2D matrix in which each row represents one thermogram of the sequence, and the column retains the N_t_ vectors of the individual thermograms. The resulting matrix is called a raster-like matrix.

For the collected data set, the 2D raster matrix, it is necessary to first calculate the mean value, which is then subtracted from the initial data set in order to normalize the data. For the matrix of normalized data, the covariance matrix **Cov** is calculated according to Equation (2):

(2) $$Cov=\frac{1}{N_{t}}XX^{T},$$

where the **X** matrix is obtained after normalization, and the covariance matrix is of the dimension N_u_·N_v_ × N_u_·N_v_.

Eigenvalues and eigenvectors are calculated by matrix diagonalization (Equation (3)):

(3) $$Cov_{D}=P^{-1}\;Cov\;P,$$

where **Cov_D_** is a diagonalized matrix with eigenvalues on the diagonal, and **P** is a matrix with eigenvectors as columns, in total it has N_u_·N_v_ eigenvectors.

The **Cov_D_** matrix needs to be rearranged in such a way that the eigenvectors are organized in descending order of magnitude. A similar change of order has to be made also in the matrix of eigenvectors **P**. For the observed data set, the eigenvector with the largest eigenvalue is the main component. In most instances, the first three to five main components contain in excess of 95% of the variance. Utilizing the main components, the data set is reconstructed, which now indicates the similarities and differences in the data set.

The main difficulty of PCT is very intense computation of diagonal matrix and eigenvalues of the 2D raster matrix. Thus, singular value decomposition (SVD) performs identical procedure as described in the equations above, but with less computation intensity. SVD is applied to normalized data to compute the decomposed matrices U, Γ and V to attain the principal components (Equation (4)):

(4) $$A=U\Gamma V^{T},$$

**A** is a 2D raster matrix consisting of N_t_ rows and N_u_ N_v_ columns, Figure 1. Matrix **A** is arranged in such a way that the columns represent temporal variations while the rows represent spatial variations. In the described structure, the columns of the matrix **U** represent a set of orthogonal statistical modes known as the empirical orthogonal functions (EOFs). The matrix **Γ** is a diagonal matrix with singular values on the diagonals. These singular values in matrix **Γ** are the eigenvalues for the corresponding eigenvectors in the matrix **V**. Columns in the matrix **V** represent the principal components or eigenvectors of the data set, and are arranged in descending order of magnitude.

Principal components, which represent time variations, have been rearranged into rows of matrix **V^T^**. The principal components and empirical orthogonal functions (EOF) acquired by using PCA have all non-zero matrix elements. 

If EOF images are depicted, the first EOF (EOF_1_) corresponds to the first most characteristic data variability, the second EOF (EOF_2_) corresponds to the second most characteristic variability, etc. The first mode is characterized by a spatially uniform field with exponential decay, which is reminiscent of the behavior of the sample without damage, from which it is not possible to determine the existence of defects, while the second mode is characterized by a non-uniform field where spatial distribution can be correlated with contrast signal, which occurs due to the defect in the sample.

**Figure 2:**
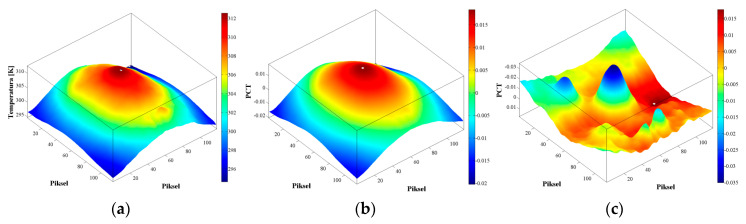
Comparison of the results obtained from raw data and using PCT: (**a**) Thermogram; (**b**) EOF_1_; (**c**) EOF_2_.

It can thus be said that EOF_1_ of PCT analysis gives an image similar to that obtained for the classical thermogram, but in the EOF_2_ a distribution is obtained that characterizes the defects embedded in the observed sample (Figure 2).

If one analyses EOFs, enhanced detectability of defects is thus assumed, but no guarantees can be given (even theoretical) that PCT can successfully isolate different information levels [36].

## 3. Materials and Methods

**Figure 3:**
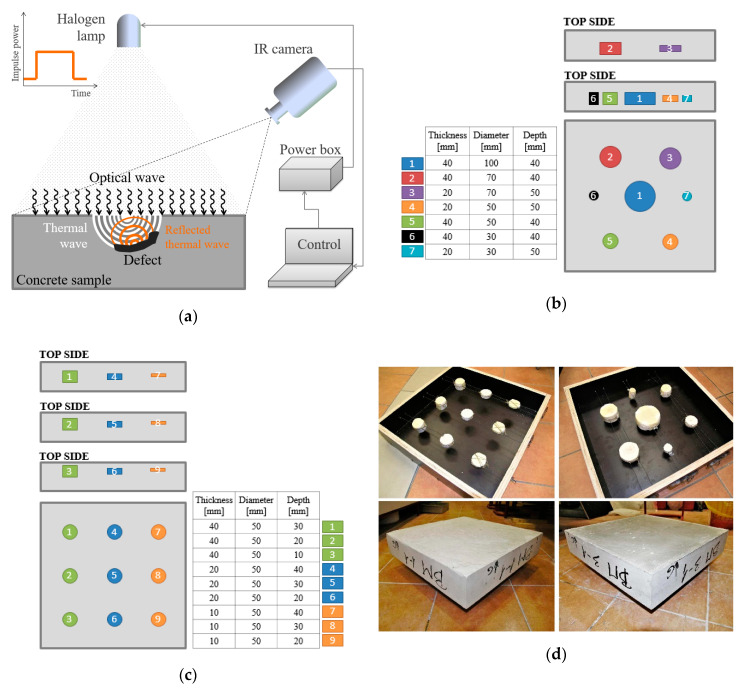
Schematic representation of: (**a**) data acquisition system; (**b**) samples BM x-1; (**c**) samples BM x-2; (**d**) photos of concrete samples and XPS defects.

The experimental setup (Figure 3a) consists of a thermal excitation unit, an infrared (IR) camera and a computer system that allows recording of digital data in real time. Figure 3a shows how long-lasting pulse of thermal excitation is introduced using halogen lamp. The optical wave emitted by the halogen lamp heats up the sample’s surface, thus inducing a thermal wave within the concrete sample. Thermal waves are reflected from the defects embedded in the samples back to the top surface and cause the deviation of the temperature field over the defects. Temperature field on the sample surface is then monitored and recorded using an IR camera. In this research, a ThermaCAM P640 IR camera (FLIR Systems, Inc., Wilsonville, OR, USA) with a spectral range 7.5–13 µm, thermal sensitivity (NETD) 60 mK and 640 × 480 Focal Plane Array (FPA) detector, was utilized to perform the experiment. As a thermal excitation source, a 1000 W halogen lamp was used and controlled by a power box and a computer using an active thermography interface. A computer was also used to control the IR camera and data collection. Pre-processing of raw data was done using the FLIR ThermaCAM^TM^ Researcher Pro 2.9 software and MatLab), where a series of 2D thermograms (720 thermograms), was converted into a 3D matrix, where rows and columns are rows and columns of the thermograms and the third dimension gives the pixel’s temporal temperature change. 

The presented research was performed using the concrete specimens with known defects, which were prepared by embedding the defect mockups using the extruded polystyrene (XPS) foam (Figure 3b,c). XPS foam was chosen to mimic the real defects because it has thermophysical properties very similar to those of air that is usually found in real defects within concrete (thermal conductivity 0.035 W/(m K), specific heat capacity 1450 J/(kg K), density 30 kg/m^3^). Also, XPS does not absorb water, which means that the properties will remain the same after concreting and conditioning of samples. Furthermore, XPS is a rigid material, which ensures that after concrete casting the defect thickness would remain as designed, and also enables easier positioning of the defects in the desired location within the specimens (having in mind the disruptive process of concreting).

Defect thickness, size and installation depth were varied so that the influence of defect’s geometry and depth could be investigated. The size and depth of the simulated defects in concrete samples tested in this research was determined with intention to simulate voids and compaction defects occurring up to the depth of concrete cover, which is critical for durability of concrete structures. The XPS foam defects simulation have been affixed and fresh concrete was casted with caution into the molds in order to obtain the planned defect depth. All the specimens were tested from both sides (top side and bottom side), in order to obtain data for as much as possible defect depths. The size of the samples was determined by the requirement that they should be relocated for storage and testing in the laboratory facilities, while on the other had they were sized to be a good representation of a real concrete structure with a representation of real defects, avoiding the influence of sample’s edges and/or interfering temperature field. Figure 3b,c give schematic representation of the samples with defects numbered and with a legend, which determines the thickness of simulated defect, its diameter and its depth from the top surface of the sample. Overall dimensions of the samples are 500 × 500 × 100 mm. The following nomenclature is used to identify specific samples (in respect to concrete type used and defect configuration): BM x-y, where “x” specifies the concrete type while “y” specifies the defect configuration in the samples, Figure 3b,c and Table 1. The defect depth is measured from the surface of the sample to the surface of the defect.

Concrete properties were also varied in order to determine its influence on te defect detectability, and for the purpose of this research, three concrete mixtures with the properties listed in Table 1 were used.

Three concrete types were used in order to replicate the three most used concrete types in real structures. Due to their different mix composition, these three concrete types vary in density, compressive strength and thermal conductivity, thermal diffusivity (due to different density and conductivity). Different thermal properties are mostly due to the different air content in concrete, since the same aggregate was used for all three mixtures, dolomite aggregate D_max_ = 16 mm. 

The samples were heated using a step heating (SH) thermography (60 min long excitation period) during which a sequence of thermograms was collected (Figure 3a). After the end of excitation period, thermograms were also collected during the cooling period, which lasted for additional 60 min. Collection of thermograms was performed at regular time intervals of 10 s during both heating and cooling periods, resulting with 720 thermograms in the sequence. The same procedure and SH duration was performed for three different thermal excitation distances (1.5, 2 and 3 m distance of the halogen lamp to the samples’ surface). Tests were performed indoors and the samples were kept in this indoor space for at least one day before testing. Also, after testing from one side (e.g. top side), samples were kept in the same environment for one day and tested from the other side (e.g. bottom side). The air temperature and relative humidity (RH) in the laboratory were monitored and kept between 18.0 and 24.0 °C and 24.3–47.5% during the tests and conditioning period of samples for all test configurations.

**Figure 4:**
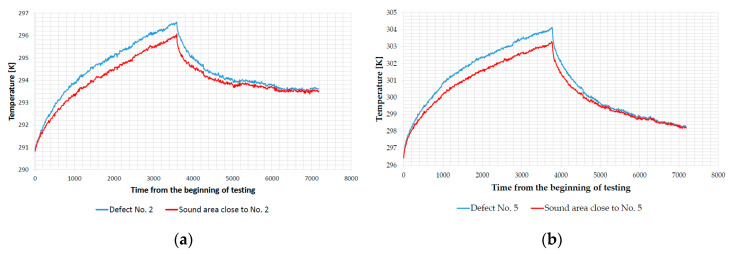
Surface temperature development: (**a**) BM 2–2–thermal excitation distance 2 m, top side, defect No. 2; (**b**) BM 3–2–thermal excitation distance 2 m, top side, defect No. 5.

The cooling period was kept at 60 min even though cooling process is slower than heating in natural conditions because preliminary tests showed that the surface temperature of the defected and sound area of concrete samples are equal and with the same rate of cooling. Figure 4 shows examples of surface temperature evolution, while for other samples and defects analogous effect was observed.

SNR was used to describe the contrast between the area of defects and neighboring sound area. SNR values were calculated for the defects in all test configurations (288 combinations of defects using optimal thermograms and a minimum of 288 additional combinations using EOFs). This allowed authors to quantify the goodness of PCT applied for defect detection in concrete compared to optimal thermograms (raw data). Additionally, SNR allowed us to quantify the impact of concrete type as well as the excitation distance on defect detectability. 

It will be shown later in the paper that defect detectability depends on the analyzed EOF and thus also SNR varies depending on the specific EOF. Due to paper length limitation, SNR is presented only for respective EOFs presented in this paper, while others can be made available if needed. 

**Figure 5:**
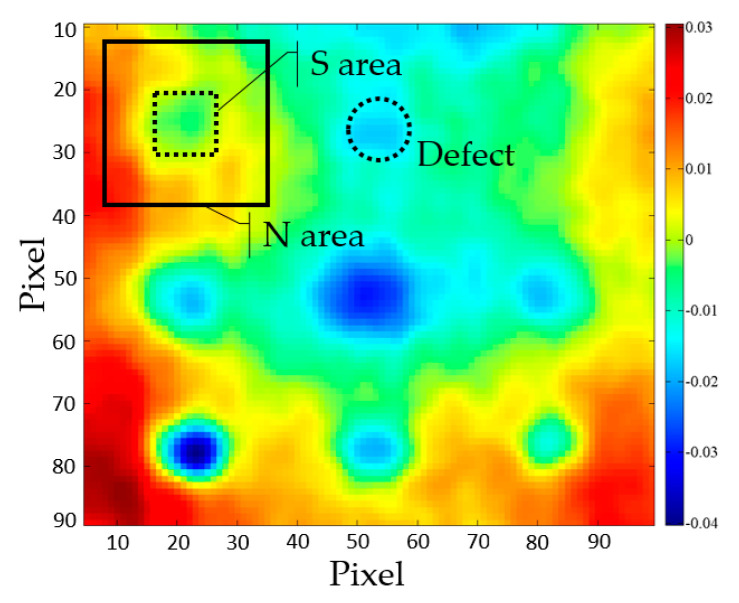
Example of areas for SNR calculation.

For the purpose of calculating the SNR, the authors adopted approach shown in [37,38], where *S* represents average values of the “signal” area on the defect, while *N* represents average values of the “noise” area around the respective defect. Equation (5) was used to calculate the SNR:

(5) $$SNR=20\times \log_{10}\left(\frac{\left|S-N\right|}{\sigma_{noise}}\right)\;\;\left[dB\right],$$

where *σ_noise_* is the standard deviation of the noise area around the defect. Here signal area was always selected to include the whole area of the defect, while the noise area encircles the defect and includes an area of half the distance to the neighboring defect (Figure 5). Square areas were used to be able to select respective pixels.

In respect to the quantitative evaluation, a defect is detected if SNR > 0 dB, but it must be noted that defects are more clearly identified if SNR value is greater.

## 4. Results

**Figure 6:**
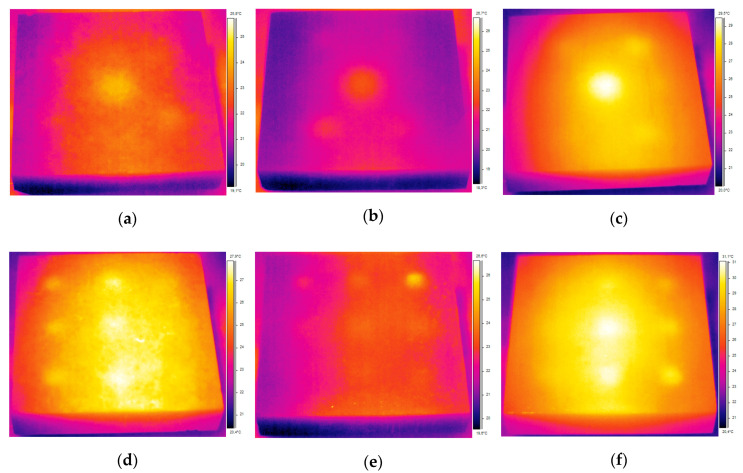
Optimal thermograms acquired for different concrete qualities: (**a**) BM 1–1 (t_opt_ = 170 s); (**b**) BM 2–1 (t_opt_ = 170 s); (**c**) BM 3–1 (t_opt_ = 100 s)—thermal excitation distance 2 m, bottom side and (**d**) BM 1–2 (t_opt_ = 120 s); (**e**) BM 2–2 (t_opt_ = 50 s); (**f**) BM 3–2 (t_opt_ = 50 s)—thermal excitation distance 2 m, top side.

Figure 6 shows the optimal thermograms acquired after the period of heating the concrete samples using 1000 W halogen lamp from the distance of 2 m. The optimal thermogram is the best thermogram chosen purely subjectively by analyzing the sequence of thermograms. 

Here, t_opt_ denotes the observation time of the optimal thermogram in seconds from the beginning of the cooling period. It is evident that some defects can be detected using these optimal thermograms, but there is a significant effect of uneven heating and reflectance, which hinder defect detection. Finding an optimal thermogram by checking every thermogram in a sequence based on visual observations can be quite tedious work.

There is a high probability that some or majority of defects will be left undetected if using optimal thermograms in NDT of concrete structures and thus post-processing of the acquired data is essential to retrieve as much information about the existence of defects as possible. Table 2 lists the SNR values for the optimal thermograms presented in Figure 6 These results support the observation obtained from qualitative analysis of optimal thermograms (raw data) and enable quantification of defect detection. 

This section gives the experimental results as well as possibilities and limitations of using the PCT as a post-processing method of the acquired data. The results are given for the cooling sequence of thermograms, which were taken after the samples had undergone the action of thermal excitation for 60 min. The EOFs presented in this paper are characteristic for individual samples and for the whole set of test results.

**Figure 7:**
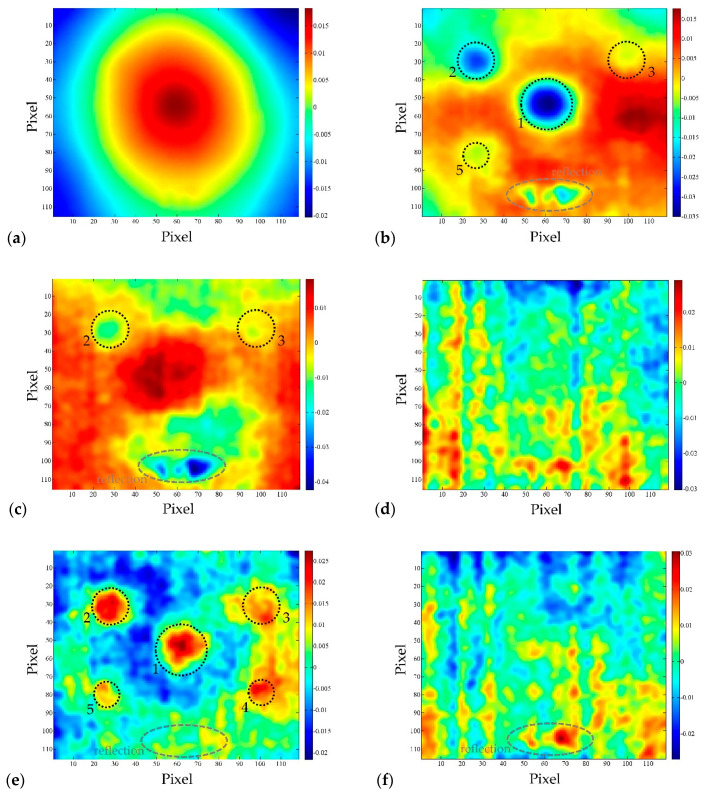
BM 1–1 bottom side, distance 1.5 m: (**a**) EOF_1_; (**b**) EOF_2_; (**c**) EOF_3_; (**d**) EOF_4_; (**e**) EOF_5_; (**f**) EOF_6_.

Figure 7 shows the first six EOFs obtained by observing the cooling sequence of the BM 1–1 sample (bottom surface), which show the characteristic results for all samples. The aim of the presentation of the first six EOFs is to show the possibility of obtaining test results using the PCT method, and the characteristic feature of the method, that in the case of certain EOFs, the existence of damage can be perfectly determined, while for the following EOF only the noise is obtained. Table 3 shows how SNR is changing for the same defect from SNR > 0 dB to SNR < 0 dB depending on analyzed EOF. This is important since it can lead to wrong conclusions about the existence of damage in the concrete samples, either leading to false positive or false negative results.

It can be seen that EOF_1_ is quite similar to the thermogram taken at the moment of turning off thermal excitation (Figure 7a) and can be said that it captured a lot of background information like non-uniform heating. On the other hand, EOF_2_ (Figure 7b) and EOF_5_ (Figure 7e) indicate that there are four and five defects, respectively, embedded in the concrete sample. 

It is also evident from Figure 7 that PCT doesn’t remove effects of reflection from the results. During the analysis of patterns on presented EOFs, it was determined that halogen lamp, which was used for thermal excitation while turned off, was still hot and radiating towards the samples and consequently its radiation was reflected from the concrete’s surface into the IR camera. Areas affected by reflection are encircled by the dashed line on the EOF-s (Figure 7). 

The reflection in this case is particularly troublesome and "dangerous", because when analyzing a single EOF, it is impossible to distinguish the reflection from the effect of the real defect, because the intensity in the area of reflection is of the same extreme as the EOF intensity occurring in the area of defect. If EOF_3_ and EOF_4_ are observed (Figure 7c and d respectively), it is not possible to detect the existence of defects in the concrete sample, in fact, the dominant influence is that of reflection in the case of EOF_3_, and noise in case of EOF_4_ and EOF_6_ (SNR < 0 as shown in Table 3). All EOFs after EOF_6_ produce noise, like the one shown in Figure 7f, and it is impossible to determine the existence of defects in concrete samples using these higher EOFs.

**Figure 8:**
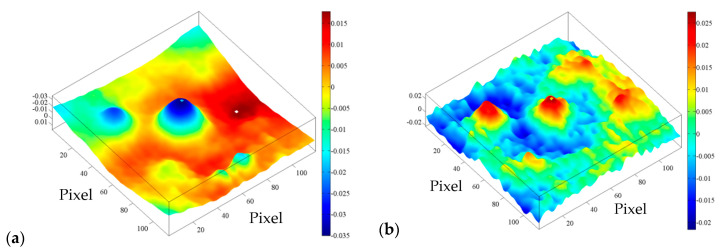
BM 1–1 bottom side, distance 1.5 m: (**a**) EOF_2_; (**b**) EOF_5_.

The SNR in EOF_5_ is slightly lower than in the case of EOF_2_ (Table 3), but the intensity above the defect area is so great that the existence of defects in the sample can be unequivocally established (Figure 8). Thus, it can be said that EOF_2_ and EOF_5_ successfully highlighted the existence of defects in the concrete sample.

**Figure 9:**
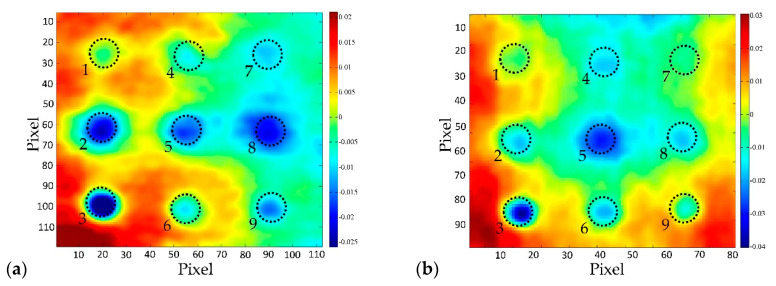

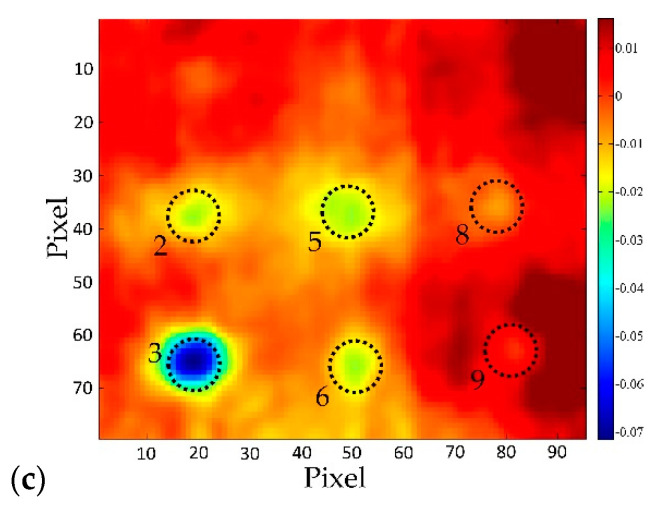
BM 2–2 top side, EOF_2_: (**a**) distance 1.5 m; (**b**) distance 2 m; (**c**) distance 3 m.

If we compare the test results of the sample BM 2–2 (top surface), it can be seen that with increasing the distance of thermal excitation from the sample’s surface (1.5 m, 2 m, 3 m), the possibility of detecting defects in concrete decreases (Figure 9). 

EOF_2_ representations are given, because there is a slight influence of uneven heating of the samples, but nevertheless, it is evident that majority of defects was detected by using PCT post-processing method. Higher SNR values (Table 4) were obtained for majority of defects for shorter excitation distances. The same phenomenon is also observed for other test configurations and respective defects. 

**Figure 10:**
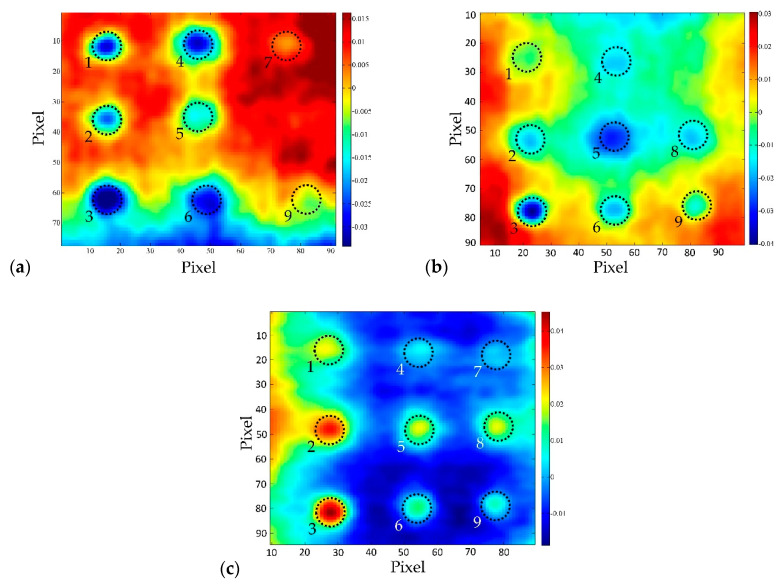
PCT (EOF_2_)—distance 2 m, top side: (**a**) BM 1–2; (**b**) BM 2–2; (**c**) BM 3–2.

Observing the influence of concrete quality on the possibility of detecting defects in samples, it can be seen that the optimal possibility of defect detection is in case of concrete BM 2-y (Figure 10, Table 5). This is explained by the fact that thermal wave cannot be transmitted to the defect and reflected back to the surface in the equal time of thermal excitation of the samples due to the low thermal conductivity of poor quality concrete. In contrast, in the case of high quality concrete and dense structure, lateral heat diffusion is dominant and it masks the existence of damage in the sample to some extent. But never the less, for all concrete types analyzed in the presented research, the results acquired using PCT post-processing method revealed defects of different thicknesses and defects located at different depths while retaining the same defect diameter.

**Figure 11:**
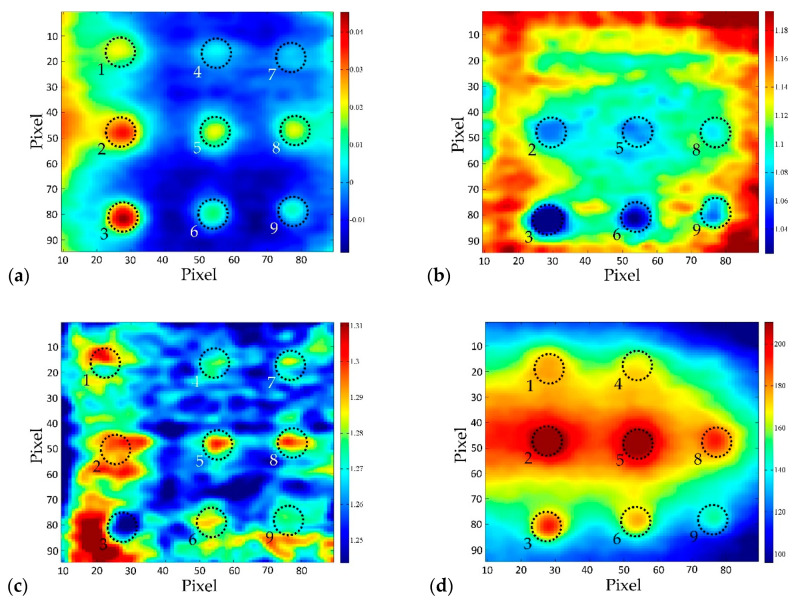
Comparison of results gained using two post-processing methods, BM 3–2, distance 2 m, top side: (**a**) PCT; (**b**) Phasegram ($f=5.764\times 10^{-4}\;\mathrm{Hz}$); (**c**) Phasegram ($f=1.441\times 10^{-3}\;\mathrm{Hz})$; (**d**) Ampligram ($f=1.441\times 10^{-3}\;\mathrm{Hz})$.

If the PCT results are compared to the results acquired by using pulsed phase thermography (PPT) as a post-processing method (Figure 11), the superiority of PCT over PPT (phase and amplitude) is evident. This is also confirmed by the SNR (Table 6) where EOF shows higher SNR values than both phasegrams and ampligrams. Even though the defect detectability depends on the frequency of the highest phase contrast and the lowest noise, in the presented research, it was not possible to find better PPT results (phasegrams) than those presented in Figure 11.

**Figure 12:**
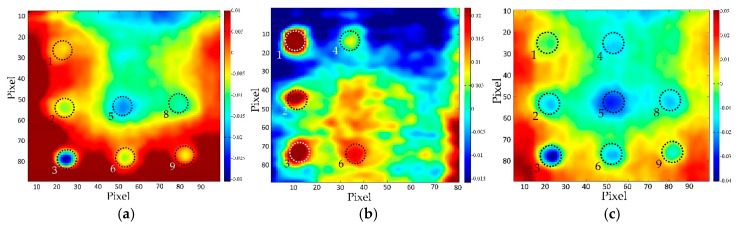
PCT, BM 2-2 distance 2m, top side: (**a**) heating & cooling sequence, (EOF_2_); (**b**) heating sequence (EOF_3_); (**c**) cooling sequence, (EOF_2_).

**Figure 13:**
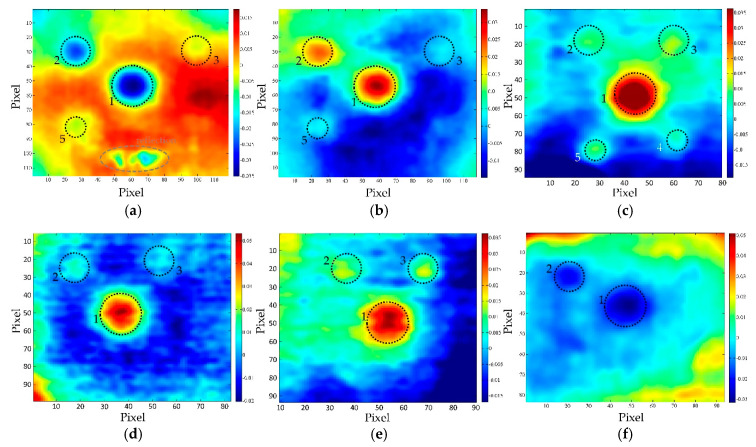
PCT, BM 1–1 bottom side: (**a**) cooling sequence, distance 1.5 m, (EOF_2_); (**b**) heating & cooling sequence, distance 1.5 m, (EOF_3_); (**c**) cooling sequence, distance 2 m, (EOF_2_); (**d**) heating & cooling sequence, distance 2 m, (EOF_4_); (**e**) cooling sequence, distance 3 m, (EOF_2_); (**f**) heating & cooling sequence, distance 3 m, (EOF_3_).

In general, it can be concluded that in case of small diameter defects, PCT analysis applied on the cooling sequence or only part of the cooling sequence proved to be a better methodology compared to an analysis of thermogram sequence acquired from heating or both heating and cooling period. There is a visible difference in the results obtained by PCT analysis (Figure 12, Figure 13) as well as in SNR values shown in Table 7 and Table 8. 

In addition to being able to detect a smaller number of defects (those of lower thickness or those deeper in concrete), there is a serious problem with the effect of reflection and uneven heating of the samples, which particularly affects the results of PCT analysis.

In the case of large defects at a relatively shallow depth, it can be concluded that there is no difference between the possibility of detecting defects by analyzing thermograms acquired during the entire sequence or only the cooling sequence.

## 5. Discussion

**Figure 14:**
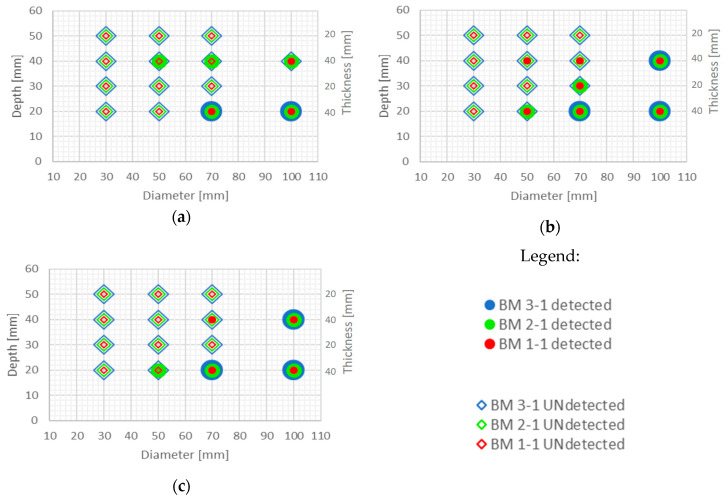
Possibility of defect detection overview for samples BM x-1 (all three concrete types) using optimal thermograms: (**a**) excitation distance 1.5 m; (**b**) excitation distance 2 m; (**c**) excitation distance 3 m.

**Figure 15:**
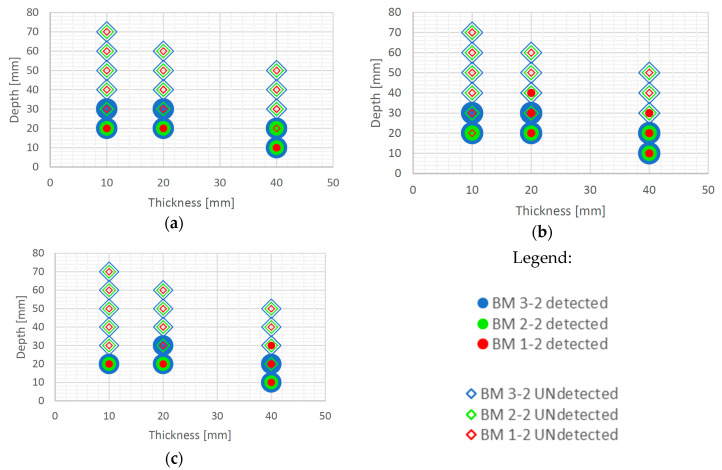
Possibility of defect detection overview for samples BM x-2 (all three concrete types) using optimal thermograms: (**a**) excitation distance 1.5 m; (**b**) excitation distance 2 m; (**c**) excitation distance 3 m.

Defect detectability using optimal thermograms with no post processing is shown in Figure 14 and Figure 15. It is evident that some of the defects are detectable (large diameter and/or shallow ones) also from the optimal thermograms, but the contrast between the defected and sound area of samples is quite low, as is SNR (Table 2). The interpretation of the existence of defects largely depends on the subjective evaluation of the person who interprets these thermograms.

**Figure 16:**
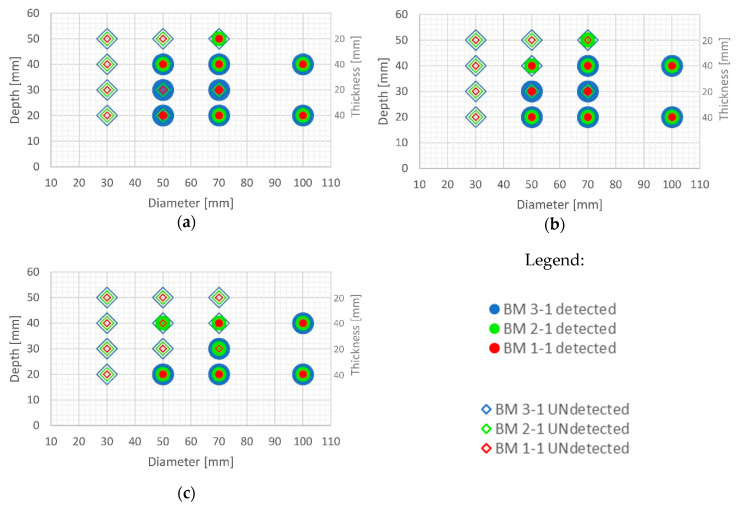
Possibility of defect detection overview for samples BM x-1 (all three concrete types) using PCT: (**a**) excitation distance 1.5 m; (**b**) excitation distance 2 m; (**c**) excitation distance 3 m.

**Figure 17:**
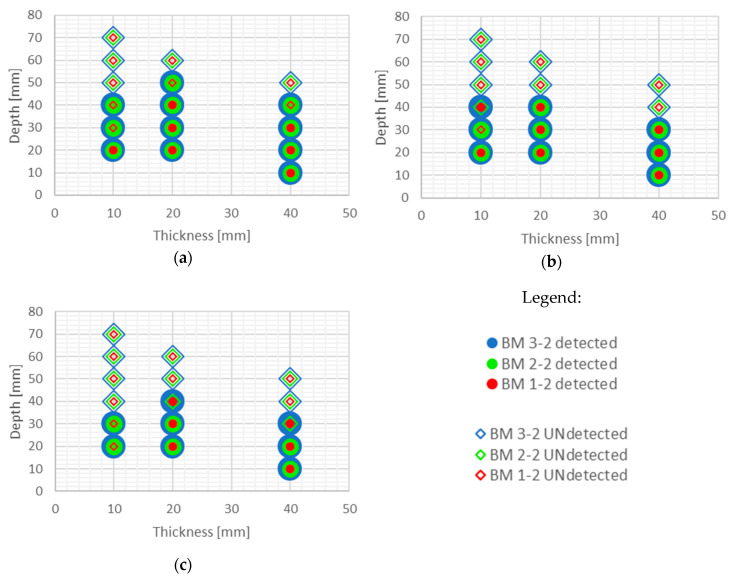
Possibility of defect detection overview for samples BM x-2 (all three concrete types) using PCT: (**a**) excitation distance 1.5 m; (**b**) excitation distance 2 m; (**c**) excitation distance 3 m.

It is evident from the thermograms shown in Figure 6 and EOFs shown in (Figure 7, Figure 8, Figure 9, Figure 10, Figure 11, Figure 12 and Figure 13) that PCT improves the detectability of defects embedded in samples under the testing conditions used in this paper. Figure 16 and Figure 17 show the defect detection results for all sample configurations and thermal excitation distances for BM x-1 and BM x-2, respectively. 

Direct comparison of SNR ratios obtained from thermograms (Table 2) and EOFs (Table 5) for test configurations BM x-2, distance 2 m, top side reveals that applying PCT significantly increases the SNR and thus the detectability of defects in concrete. For example, the SNRs obtained from optimal thermograms (Table 2) are in the range from −4.22 dB up to 10.46 dB (with 12 SNR values < 0 dB), while SNRs obtained from EOFs for the same test configurations are in the range from −4.44 dB up to 24.08 dB but with only 1 SNR value < 0 dB. 

Large defects (100 mm in diameter) can be detected at a depth of 40 mm using PCT in case of all concrete qualities and excitation distances used in this research. Defects of 50 and 70 mm in diameter and up to 40 mm in depth are also detectable with high certainty if they are at least 20 mm thick and if there is enough energy transferred into the specimen (at 1.5 m and 2 m distance). This is encouraging since, honeycombs and voids in concrete structures occur mainly up to concrete cover depth and are of large diameter and thickness. Thicker defects are also more likely to be detected compared to lower thickness defects even though defects are of the same diameter (Figure 16 and Figure 17). It is evident that the number of detected defects is decreasing with increased distance of thermal excitation from the specimen surface. This can lead to the conclusion that the energy inserted into the specimen is of crucial importance to be able to detect defects and that PCT is able to overcome issues related to non-uniform heating of concrete samples. The defect detection could be enhanced for concrete structures thermally excited at larger distances if more energy is inserted into the structure using more powerful heat source. 

Some defects, which were not visible in the sample with lower quality concrete BM 1-y, became visible for BM 2-y and BM 3-y concretes, especially those of lower thickness (i.e. 10 mm) and those embedded deeper into samples (Figure 16 and Figure 17). The detectability of defects also depends on all three geometrical parameters of defects, their diameter, thickness and depth of embedment (Figure 16 and Figure 17). As can be seen (Figure 16), PCT did not manage to detect the smallest defects in samples BM x-1 (those of 30 mm in diameter) and the specimens at largest depths.

## 6. Conclusions

It can be concluded from the presented research that PCT is an effective post-processing method to improve defect detection in concrete structures. This paper showed that PCT applied to a sequence of thermograms can increase the SNR of defects compared to optimal thermograms in the case of the presented testing methodology. Even though a majority of defects were detected (even those with relatively low thickness), the results are still to be considered with care since the effects like reflection and noise can have an influence.

It was shown in the presented research that for all concrete types analyzed, the results acquired using PCT post-processing method revealed defects of different thicknesses (10–40 mm) and defects located at different depths (up to 40 mm) with 1000 W halogen lamp as a thermal excitation at maximum 3 m distance.

PCT proved to be quite valuable for detecting small defects in concrete samples (50 mm in diameter), but interpretation of EOFs has to be performed with care due to the possible influence of reflection in certain EOFs. The reflection can be identified by analyzing several EOFs where defects pattern will change slightly while reflection pattern will change significantly. It has to be highlighted here that the main difficulty of PCT is very intense computation, which means quite powerful computers are needed, especially when one uses high resolution IR camera.

It was confirmed in this research that excitation source (halogen lamp) distance from the samples influences the detectability of defects since less energy is entering the samples with greater distance making defects harder to detect, but at the same time samples are heated more uniform which is beneficial for defect detection. SNRs obtained for excitation distance of 2 m were lower than those obtained for 1.5 m distance, but never the less 2 m distance enabled more uniform heating of samples and would also enable heating of larger surface area while testing in-situ.

The quality of concrete also affects the possibility of defect detection, with the obvious difference in the possibility of detecting defects in samples marked BM 1-y (compressive strength 18.93 MPa), compared to samples BM 2-y and BM 3-y strengths of 40.99 MPa and 89.05 MPa), for which it is generally possible to detect a larger number of defects in this way. These defects are also more pronounced (have better contrast and larger SNR) compared to the undamaged part of the sample. In the case of a better quality concrete, it is possible to detect defects at greater depths, as well as defects of smaller thickness compared to concrete of poor quality. The reason for better defect detectability in BM 2-y and BM 3-y samples is higher thermal conductivity of concrete out of which the mentioned samples are made. This higher thermal conductivity allows faster thermal wave propagation to the defect, compared to lateral heat propagation which to some extent masks the existence of the defect. Although concrete’s thermophysical properties affect the test results, it was shown in this research that detection of defects is nevertheless feasible if the measurement setup is adapted accordingly.

SNRs obtained from EOFs were generally a bit higher than those obtained from phasegrams and ampligrams for the same test configurations, which means that for defect detection in concrete, PCT has comparable results to PPT, even though it has some limitations in defect quantification potential, computation intensity and the influence of reflections. It is noted that the results and conclusions of this research are only valid for the environmental conditions and the test conditions discussed.

Additional research needs to be performed in order to implement the proposed methods for inspection of real reinforced concrete structures. These could include the variation of thermal excitation periods (longer excitation with the same power of the halogen lamp) or using more powerful halogen lamps to achieve shorter excitation periods making the method more viable for in-situ testing. One could investigate the existence of optimum combination of excitation source energy input and the excitation period. There is also a possibility to explore improved PCA techniques developed in the field of carbon fiber reinforced polymers and metals and their applicability for defect detection in concrete structures. These techniques include Stable principal component pursuit (SPCP), Weighted PCT, Sparse PCT, Generative PCT, etc.

## Figures and Tables

**Table 1:** Physical and mechanical properties of three concrete types used.

Concrete Properties	BM 1-y	BM 2-y	BM 3-y
Compressive strength [MPa]	18.93	40.99	89.05
Thermal conductivity [W/m·K]	1.73	2.21	2.80
Thermal diffusivity [×10^−7^ m^2^/s]	8.0	9.1	1.1
Density [kg/m^3^]	2194	2383	2546
Air content [%]	10.5	4.6	1.4
Emissivity [–]	0.928	0.957	0.958

**Table 2:** SNR values obtained from thermograms presented in Figure 6.

Test Configuration	Defect No.	SNR [dB]		
		BM 1–1	BM 2–1	BM 3–1
BM x-1 Distance 2 m Bottom side	1	13.06	16.03	17.91
	2	6.02	8.87	8.20
	3	−2.77	4.81	0.92
	4	−9.54	2.69	−0.67
	5	−1.02	7.43	1.24
	6	−2.36	−4.86	−5.58
	7	−3.03	−9.25	−3.23
		**BM 1–2**	**BM 2–2**	**BM 3–2**
BM x-2 Distance 2 m Top side	1	5.04	−2.92	−1.82
	2	6.74	−0.83	6.02
	3	7.26	10.46	8.52
	4	3.97	−0.83	−1.09
	5	3.10	−1.58	6.53
	6	4.44	4.81	6.62
	7	−0.42	−0.83	−0.42
	8	−4.22	−3.23	4.44
	9	−3.52	2.69	3.52

**Table 3:** SNR values obtained from EOFs presented in Figure 7.

Test Configuration	Defect No.	SNR [dB]					
		EOF_1_	EOF_2_	EOF_3_	EOF_4_	EOF_5_	EOF_6_
BM 1–1, distance 1.5 m, bottom side	1	−2.50	15.27	−3.10	−6.02	12.57	−9.54
	2	−3.52	12.04	5.46	−12.04	16.03	−12.04
	3	−3.81	3.52	2.92	−7.28	8.57	−3.52
	4	−2.99	−1.58	−3.19	−6.24	5.74	−4.61
	5	−5.60	4.86	−8.52	−6.65	8.52	−8.30
	6	−4.08	−16.90	−18.06	−3.99	−10.63	−8.94
	7	−3.47	−5.58	−5.42	−6.62	−1.87	−3.52

**Table 4:** SNR values obtained from EOFs presented in Figure 9.

Test Configuration	Defect No.	SNR [dB]		
		Distance 1.5 m	Distance 2 m	Distance 3 m
BM 2–2, Top side, EOF_2_	1	14.81	9.95	−3.10
	2	25.58	14.61	7.96
	3	27.51	18.49	25.42
	4	8.52	13.62	−2.27
	5	23.52	12.04	10.88
	6	19.50	14.78	12.96
	7	22.92	6.02	−2.92
	8	23.91	13.06	4.12
	9	24.22	14.93	2.92

**Table 5:** SNR values obtained from EOFs presented in Figure 10.

Test Configuration	Defect No.	SNR [dB]		
		BM 1–2	BM 2–2	BM 3–2
BM x-2 Distance 2 m Bottom side	1	24.08	9.95	6.72
	2	13.98	14.61	17.31
	3	23.23	18.49	20.83
	4	19.28	13.62	4.86
	5	8.52	12.04	12.87
	6	6.02	14.78	14.54
	7	2.50	−6.02	2.28
	8	−4.44	13.06	10.88
	9	1.34	14.93	9.54

**Table 6:** SNR values obtained from images presented in Figure 11.

Test Configuration	Defect No.	SNR [dB]			
		PCT	Phasegram $f=5.764\times 10^{-4}\;\;\mathrm{Hz}$	Phasegram $f=1.441\times 10^{-3}\;\mathrm{Hz}$	Ampligram $f=1.441\times 10^{-3}\;\mathrm{Hz}$
BM 3–2 Distance 2 m Top side	1	6.72	−4.08	3.52	4.49
	2	17.31	6.38	5.68	10.93
	3	20.83	11.70	4.35	11.90
	4	4.86	−11.48	3.03	3.10
	5	12.87	4.86	9.12	11.06
	6	14.54	7.60	4.86	11.59
	7	2.28	−2.41	3.52	−0.33
	8	10.88	3.52	8.15	4.01
	9	9.54	6.72	−14.81	2.24

**Table 7:** SNR values obtained from images presented in Figure 12.

Test Configuration	Defect No.	SNR [dB]		
		Heating & Cooling Sequence, (EOF_2_)	Heating Sequence, (EOF_3_)	Cooling Sequence, (EOF_2_)
BM 2–2 Distance 2 m Top side	1	1.58	21.29	9.95
	2	10.37	16.69	14.61
	3	23.81	18.82	18.49
	4	−2.11	7.20	13.62
	5	11.29	−3.52	12.04
	6	9.54	2.50	14.78
	7	−3.19	−7.96	−6.02
	8	2.50	−4.61	13.06
	9	7.20	−6.02	14.93

**Table 8:** SNR values obtained from images presented in Figure 13.

Test Configuration	Defect No.	SNR [dB]					
		Distance 1.5m		Distance 2m		Distance 3m	
		Cooling Sequence, EOF_2_	Heating & Cooling Sequence, EOF_3_	Cooling Sequence, EOF_2_	Heating & Cooling Sequence, EOF_3_	Cooling Sequence, EOF_2_	Heating & Cooling Sequence, EOF_3_
BM 1–1 Bottom side	1	15.27	19.08	20.56	13.98	12.46	4.44
	2	12.04	12.24	3.52	6.02	1.94	1.94
	3	3.52	1.16	2.50	1.94	4.52	−1.58
	4	−1.58	−9.54	4.86	−3.52	−6.02	−6.02
	5	4.86	2.50	12.04	−3.65	−2.05	−2.28
	6	−16.90	−4.61	−6.02	−8.20	−7.96	−4.44
	9	−5.58	−7.96	−3.52	−10.70	−3.52	−4.86
